# The Chinese Government’s Response to the Pandemic: Measures, Dynamic Changes, and Main Patterns

**DOI:** 10.3390/healthcare9081020

**Published:** 2021-08-08

**Authors:** Yuxi He, Maorui Li, Qixi Zhong, Qi Li, Ruishi Yang, Jing Lin, Xiaojun Zhang

**Affiliations:** 1 School of Economics and Management, Fuzhou University, Fuzhou 350108, China; 071807212@fzu.edu.cn (Y.H.); 071907110@fzu.edu.cn (M.L.); 071907132@fzu.edu.cn (Q.L.); 071907127@fzu.edu.cn (R.Y.); 071907125@fzu.edu.cn (J.L.); 2 School of Public Administration and Policy, Renmin University of China, Beijing 100872, China; 071707209@fzu.edu.cn; 3 Fujian Emergency Management Research Center, Fuzhou University, Fuzhou 350108, China

**Keywords:** COVID-19, official government Weibo text, pandemic governance measures, dynamic changes, pandemic governance patterns

## Abstract

(1) Background: The governance measures that governments deploy vary substantially across countries and even within countries; there is, however, limited knowledge of the responses of local governments or from different areas in the same country. (2) Methods: By using grounded theory and an automatic text processing method, this study analyses the pandemic governance measures, the pandemic governance pattern, and possible factors across 28 provinces in mainland China based on the text of 28 official provincial government Sina microblogs dating from 20 January to 1 July 2020. (3) Results and discussion: The provincial pandemic governance patterns in China are divided into a pathogen-control pattern, a diagnosis and treatment consolidation pattern, a balanced promotion pattern, a quick-adjustment response pattern, and a recovery-oriented pattern. The pandemic severity, economic development, public health service, and population structure may all have an impact on pandemic governance measures. (4) Conclusions: The conclusions of this study may help us to reconstruct governance systems related to global public health emergencies from the perspective of normalisation, as well as providing important clarification for management and a reference for countries seeking to curb the global spread of a pandemic.

## 1. Introduction

The COVID-19 pandemic emerged in December 2019 and spread globally to become a worldwide health threat by March 2020. As the most serious public health emergency in recent decades [1,2], COVID-19 has caused a major health crisis. As of the time of writing, the WHO reports 174,061,995 confirmed cases and 3,758,560 deaths worldwide [3]. The COVID-19 pandemic is not just a health crisis but rather a crisis that has affected every sector, including the economy, politics, and society. In fact, it has prompted the worst global recession in nearly a century [4]. The WHO anticipates that the COVID-19 pandemic will have a lengthy duration [5] and has called upon all countries, international organisations, the private sector, charities, and individuals to contribute to containment efforts [6].

The outbreak of COVID-19 has prompted governments around the world to take different measures in the fight against the pandemic. Existing studies have reported on research conducted about governmental responses in different countries. From the policy perspective, the governmental responses to COVID-19 mainly comprise four parts. We categorised the COVID-19 governance measures and aggregated the governmental response data into a series of indices to describe the evolution of government responses to COVID-19. Siddik and others developed a multidimensional index for measuring the extent and progress of the government’s economic response among various countries, which indicated stronger economic responses in Chile, Switzerland, Croatia, Sweden, and the Netherlands [7]. Hale et al. created a series of indexes to measure the extent of the overall government response, government containment efforts, and government health response, as well as the stringency of the governmental response, then used them to track the evolution of governmental responses in nearly all countries, territories, and regions of the world [8]. Based on these categories of government measures and indexes, Berardi and others analysed the government’s response in Italy and its impact on both health and non-health outcomes [9]. Specific factors shaped pandemic governance measures. Toshkov et al. proposed several dimensions, including governance capacities, especially in the health sector, societal trust, government type, and party preference, to explain variations in governmental responses among EU member states, the UK, Switzerland, Iceland, and Norway [10]. To enrich the large-n comparative studies focusing on state-level responses to COVID-19, Caoano et al. tracked the differences in governmental responses between China, Italy, Singapore, South Korea, Canada, Hong Kong, Turkey, Israel, the USA, and Sweden, and then identified several factors, including the policy capacity, lessons from past similar experiences, and the nature of national leadership, as possible determinants [11]. In eight South American countries, the speed of the governments’ stringent measures was accelerated by fiscal expenditure on health, local government capacity, and pressure on the health system [12]. Additionally, governmental responses in most countries in the world are shaped by factors such as the median age of the population, the number of hospital beds per capita, the number of total COVID-19 cases, GDP, and progress in scientific research [7,13]. The third policy perspective category of governmental responses to COVID-19 is the impact of pandemic governance measures on public health, society, and economics. Economically, governmental intervention measures, including fiscal stimulus [14], containment and closure [15], risk communication, and testing and quarantining measures [16], probably resulted in less market volatility and higher recovery. For public health, statewide social distancing measures and travel bans were associated with a decreased COVID-19 case growth rate in 142 countries [17,18]. The implementation of containment measures and government announcements regarding the wearing of masks by the public, proved effective in reducing the COVID-19 mortality rate in 162 countries, including the US [19,20]. In terms of social psychology, risk communication has been effective in alleviating the social anxiety, fears, and uncertainty caused by the pandemic [21]. Last but not least, we explored the correlates of governance measures’ effectiveness. In Bangladesh, governmental coercive capacity, effective societal autonomy, and the legitimacy of the state were significantly affected by the effectiveness of lockdown measures [22]. Additionally, public awareness is positively associated with the effectiveness of government interventions through changes in people’s behaviour [23].

The governance measures deployed by governments vary substantially across countries and even within countries [8]. The situation in India emphasised that state and local governments need to adjust governmental responses based on local needs instead of indiscriminately implementing one-size-fits-all governance measures imposed by the central government [24]. However, previous research has mainly focused on the governmental responses of state-level governments, while ignoring the role of local governments or the response in different areas of the same country. This has created a need to research the differences between governments’ responses to COVID-19 and consider whether the current status of crisis response could be improved. China’s vast scale and the varying severity of the COVID-19 pandemic across the country have rendered the governance situation complex. 

This study aims to gain insight into the different pandemic governance measures deployed by Chinese provincial-level governments, the typical pandemic governance patterns used, and the influence factors involved. The remainder of this study is organised as follows: Section 2 describes the data source and analysis process, Section 3 reports the main results, Section 4 discusses the theoretical value and policy implications, and Section 5 concludes the paper.

## 2. Materials and Methods

### 2.1. Data Source and Preprocessing

Sina Microblog (also called Weibo) is the largest microblog service provider in mainland China; essentially, it is China’s equivalent to Twitter [25]. Government agency accounts have been a public administration tool to keep netizens informed [26]. Weibo data relating to the COVID-19 pandemic from 20 January to 1 July 2020 were retrieved from 2353 government agency accounts, including accounts from provincial, municipal, and county-level governments in mainland China; each represents the official information release for a given region. The engagement data (Weibo release time, Weibo website, and content) of posts from government agency accounts were extracted to evaluate government responses to COVID-19. After removing duplicate and missing data, 2,000,878 effective microblogs were left. A total of 64,544 microblogs published by the official microblog platforms of various regions were collected. Except for a few regions, the number of Weibo blogs posted in other regions exceeded 16,000. Qinghai Province, Tibet Autonomous Region, and Ningxia Hui Autonomous Region were not used as targets of this study because they released fewer Weibo posts than the other provinces and often released no Weibo posts for several days, which was not conducive to our research on daily pandemic governance measures in these three provinces. 

To improve the representativeness of the text data, we removed short text and special characters using the “re” toolkit in Python. Then, we deleted data with an NA value in the “Title” field to ensure the validity of the statistical analysis. The “jieba” package in Python was used to segment Weibo text. Finally, we divided the types of words used into 7 categories, including nouns (including names, place names, and organisation names), English words, verbs, and adverbs.

### 2.2. Data Analysis 

Considering the policy implications of the text found, we attempted to link the data to the relevant government measures. The existent and relevant research laid a good foundation for this. The Oxford COVID-19 Government Response Tracker (OxCGRT) collects data from publicly available sources such as news articles and government press releases and briefings, providing a systematic cross-national, cross-temporal catalogue of measures to help us understand government responses. As Hale et al. describes, the OxCGRT indicators include 4 categories (containment and closure, economic response, health systems, and miscellaneous) and 18 subcategories, which are also listed in the coding book [27]. Cheng et al. created a government policy activity index ranging from 0 to 100 (https://www.coronanet-project.org/, accessed on 1 August 2021) and used an artificial intelligence company to identify the press releases relating to COVID-19 and extract information on the dates and scales of the implementation of a wide range of public health measures—e.g., school or restaurant closures, as well as mobilisations of volunteers, nurses, and doctors. After this, the results obtained from the web sources as well as those found manually were coded in the CoronaNet dataset [28]. Zheng et al. created the HIT-COVID tracker, which follows 21 nonpharmaceutical interventions in 142 countries and is structured in two levels, national or regional, based on official sources and secondary sources. The authors used two levels of intervention: partial or strong [29]. Larrive et al. coded a structured dataset of nonpharmaceutical interventions divided into 8 themes and 63 categories in 56 countries: the Complexity Science Hub COVID-19 Control Strategies List (CCCSL) [30].

The grounded theory and automatic text processing methods were used to code microblog text. The grounded theory is based on the development of a set of categories and classifications that are interrelated to form an integrated framework for interpreting or predicting a phenomenon [27,31]. Automatic text analysis uses a series of technologies for machine learning analysis and the mining of large volumes of text data using computers [32,33]. By viewing the text as a database composed of many words, it is possible to detect hidden topics by analysing which words frequently appear together and which words appear more often. With the guidance of grounded theory, a three-phase coding process was used; the first step was open coding, which was followed by axial and selective coding [34]. To start with, automatic text analysis and statistical methods were used in this research. The main algorithm used in the topic model is LDA (Latent Dirichlet Allocation) [35]. This method significantly improves the efficiency of the manual analysis of large volumes of text, similarly to dimensionality reduction in factor analysis. It enables the user to focus on the topic of each document and provides this information in the form of a probability distribution. In the axial coding phase, to form more complex clusters of categories and subcategories, the categories established in the open coding phase are refined and reorganised to specify the properties and dimensions of a category [36]. After extracting their topics (distribution) by analysing some documents, topic clustering or text classification can be carried out according to the topic involved (distribution). In the selective coding phase, the core category is identified to elucidate the essence of the phenomenon [34]. The core categories are characterised by several properties, including a high frequency, a considerable degree of abstraction, and extensive connections with all other categories and codes [37]. These categories can completely show the main information in the microblogs, thus offering a clear insight into the measures taken by governments. 

The microblog text was coded according to our coding book. The coding book (which lists the vocabulary contained in each category) is provided in Table 1. The opening coding index contains the keywords from the microblog text, the axial coding index can be seen as the governments’ measures, and the selective coding index is used to elucidate the essence of the measures taken. If the microblog text contains vocabulary used for two topics, it is worth considering that the microblog may belong to the interactive theme category.

**Figure 1:**
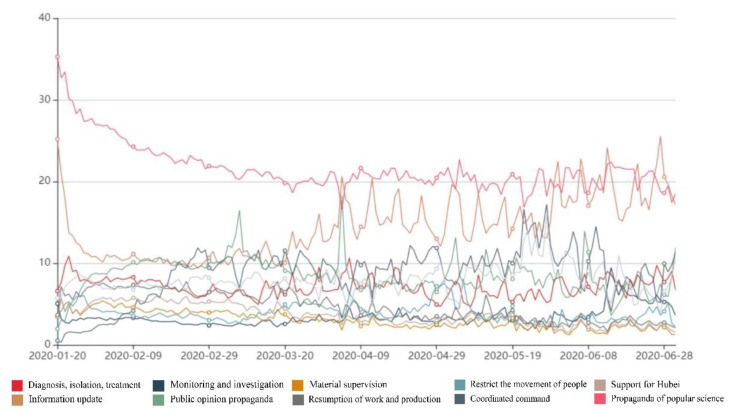
Dynamic evolution of pandemic governance measures in China.

Any words that are not semantic, as well as the URL codes and emoticons in all the microblogs, are filtered out; moreover, keywords are automatically matched and word percentage statistical analyses are performed using computer programs. Our data came from 28 provinces from 20 January to 1 July 2020. We did not take the number of microblogs made as the unit of measurement, but instead used how often a certain type of keyword appeared. This enabled us to determine whether the account was more inclined to publish a certain kind of information or pay more attention to certain governance measures. We recorded the time of publication, the region, and the corresponding categories of word frequency, then analysed and displayed them through the total word frequencies and a data map (please see Figure 1).

Finally, the 23 pandemic governance measures implemented by provincial governments from 20 January to 1 July 2020 were used as the training set for a K-means algorithm, and five types of portfolios of measures were summarised by the clustering method. In this study, we also analysed the similarities and differences of specific provincial-level government responses, which can be divided into five types of governance patterns based on the configuration status.

## 3. Results

To better understand the dynamic evolution of pandemic governance measures in Chinese provincial governments, we used the 23 pandemic governance measures implemented by provincial governments from 20 January to 1 July 2020 as the training set for a K-means algorithm, and five types of portfolios of measures were summarised by our clustering method. The figures show the portfolios of measures that the government implemented every day from 20 January to 1 July 2020 in 28 provinces in mainland China. Among them, the red bar represents balanced promotion measures, which relates the ratio of the various measures to their relative dispersion; the green bar represents forced specification measures, which means that the government put emphasis on command, coordination, security, and regulatory measures; the yellow bar represents health promotion measures, which means that the government focused on medical treatment and personnel mobility measures; the blue bar represents recovery-oriented measures, which means that the government focused on measures concerning resuming work, resuming production, and resuming school; the teal bar represents information support measures, which means that the government focused on measures related to information.

### 3.1. Overview of the Dynamic Evolution of Pandemic Governance Measures

#### 3.1.1. Comprehensive National Level

Figure 1 shows the dynamic evolution of pandemic governance measures in China on a comprehensive national level from 20 January to 1 July 2020. Propaganda of popular science (25.8%) was the most important measure, showing a slow downward trend from 20 January to 22 March and remaining stable in the subsequent period. Information updates accounted for 17.2%, showing a rapid downward trend from 20 January to 23 January, remaining relatively stable until 19 March, then showing an upward trend from 20 March to 1 July. The resumption of work and production measures accounted for 10.3%, showing a continuous upward trend from 20 January to 28 May and then a slow decline from 29 May to 1 July. Public opinion propaganda measures accounted for 10.2%, showing a gradual upward trend from 20 January to 8 March and a slow downward trend from 9 March to 1 July. Coordinated command (9.5%) measures remained stable overall, with only minor changes. Beyond these, there were also diagnosis, isolation, and treatment measures. In terms of the overall relative proportions, these measures occupied the middle of the use of all measures, at 8.5%. Theses measure showed a slow downward trend from 20 January to 13 March, an upward fluctuation from 14 March to 18 April, a small decline from 19 April to 8 May, then a gradual rise until they reached a peak. Measures based on restricting the movement of people (4.6%) maintained a relatively stable trend from 20 January to 6 May and showed a gradual downward trend from 7 May to 1 July. Support for Hubei measures (5.6%) showed a slow downward trend throughout the entire observation period. Meanwhile, material supervision (4.3%) remained stable throughout the entire observation period. 

**Figure 2:**
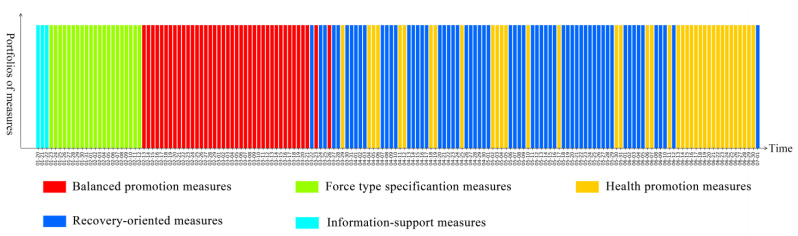
The portfolios of measures implemented in mainland China.

Figure 2 shows the portfolios of measures taken every day on the comprehensive national level in mainland China. The measures taken can be divided into three phases. The first phase is the positive response period of China’s pandemic governance (from 20 January to 12 February 2020). During this period, the pandemic broke out across the country and there was an increase in pressure on pandemic governance. Therefore, the government mainly adopted force-type specification measures to effectively control the spread of the epidemic and respond to the original crisis. The second phase was the period of initial control of the pandemic (from 13 February to 26 March 2020). During this period, the spread of the pandemic across the country was effectively controlled. From mid-March, the number of new cases every day was controlled to within single digits, while the epidemic prevention and control achieved important phased results. Therefore, the government began to adopt a variety of balanced promotion measures to further control the spread of the pandemic and promote social recovery in an orderly manner. The third phase was the period of recovery and consolidation (from 27 March to 1 July 2020). The country’s pandemic situation in this period was basically stable, and the pandemic governance entered a normalised stage. During this period, the government mainly adopted recovery-oriented measures and health promotion measures to promote social recovery and development, as well as to consolidate the results of pandemic governance to prevent a second outbreak of the pandemic, so as to effectively deal with the secondary crisis.

#### 3.1.2. Provincial Level

**Figure 3:**
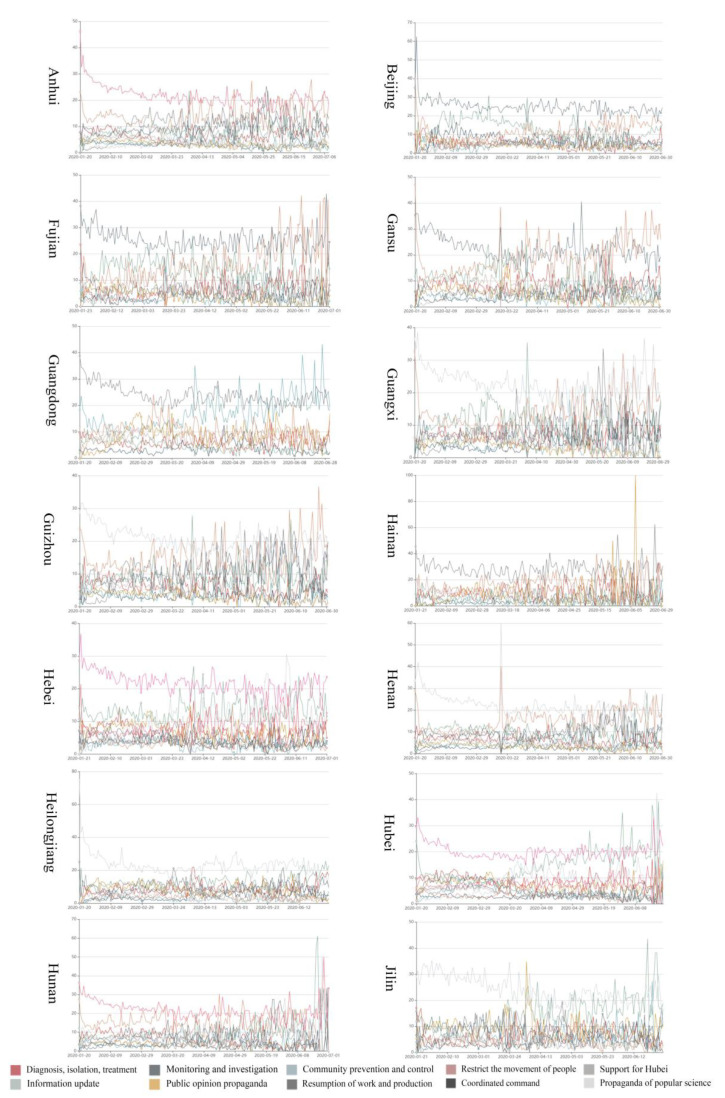

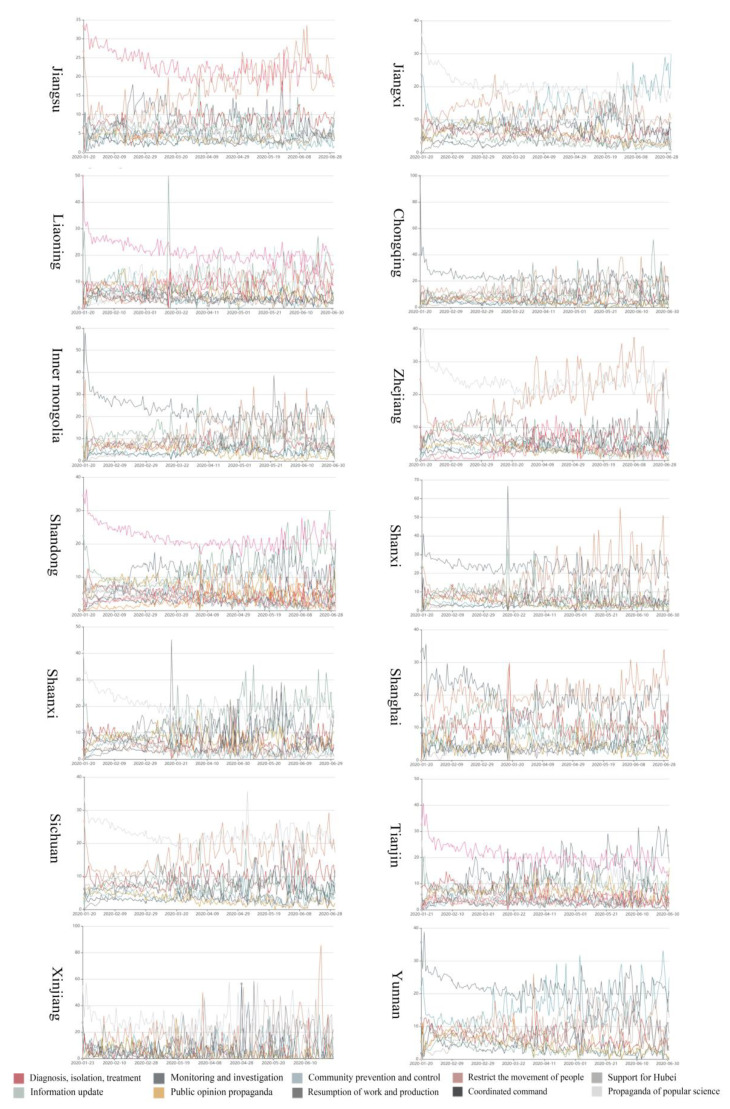
Overview of the dynamic evolution of pandemic governance measures in China.

Figure 3 shows the dynamic evolution of pandemic governance measures. The frequency was over 3% from 20 January to 1 July in 28 provinces of mainland China. There are 10 measures in Table 1 for which percentage statistics were over 3%, including information update, propaganda popular science, public opinion propaganda, diagnosis, isolation and treatment, the resumption of work and production, command and coordination, support for Hubei Province, restricting the movement of people, community prevention and control, and monitoring and investigation. This indicates that these were the main measures the provincial government took. 

There are some obvious differences between provinces, relating to different measures being deployed at the same time and the same measures happening at different times. In general, Shanghai City, Zhejiang Province, Guangdong Province, and Hainan Province, among others, placed more weight on information update measures. Hainan Province, Fujian Province, Beijing City, Jilin Province, and Zhejiang Province, among others, paid more attention to propagandist popular science. Fujian Province, Beijing City, Jiangxi Province, Shanghai City, and Hunan Province, among others, put more emphasis on public opinion propaganda, while Anhui Province, Hebei Province, Hubei Province, Jiangsu Province, and Liaoning Province, among others, paid more attention to diagnosis, isolation, and treatment. Shanghai City, Yunnan Province, Inner Mongolia, Beijing City, and Jilin Province, among others, were more concerned with the resumption of work and production, and Chongqing City, Hunan Province, Liaoning Province, Guizhou Province, and Tianjin City, among others, focused more on command and coordination. Hubei Province, Jiangxi Province, Jiangsu Province, Tianjin City, and Liaoning Province put more emphasis on support for Hubei Province, while Shanghai City, Hainan Province, Inner Mongolia, Sichuan Province, and Shandong Province, among others, paid more heed to restricting the movement of people. Finally, Beijing City, Jilin Province, Hubei Province, Liaoning Province, and Shandong Province, among others, put more emphasis on community prevention and control.

### 3.2. Pandemic Governance Patterns in Chinese Provincial Government

This study also analysed the similarities and differences of specific provincial-level government responses, created five types of policy distribution models, and named them based on the configuration status of five typical measures.

#### 3.2.1. Pathogen-Control Pattern

**Figure 4:**
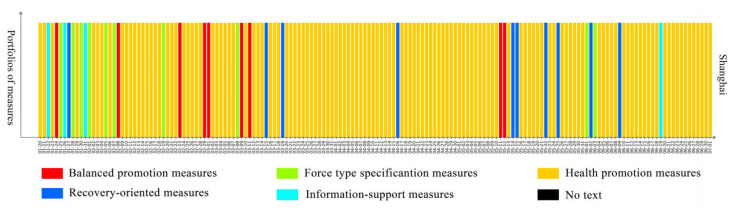
Pathogen-control pattern.

Figure 4 shows that the government response to the pandemic in Shanghai was quite different from the response of other areas. During the study period, balanced promotion measures were the main measures taken for 12 days around 1 March and 1 May; force-type specification measures were the main measures taken for 14 days and were concentrated in the early stage of the pandemic; health promotion measures were the main measures taken for 115 days; recovery-oriented measures were the main measures taken for 12 days; information-support measures were the main measures taken for 11 days and were concentrated in the early stage of the pandemic. As a central city in China and the “gateway” to the outside world, Shanghai focused on publishing policies related to primary crisis management in the whole process of public health crisis management, focusing on the pandemic situation and improving the public’s perception of prevention and control measures; thus, it managed to effectively promote crisis management and stabilise the social order. Shanghai raised the public’s awareness of the need for the prevention and control of the epidemic, thereby effectively advancing the prevention and control of COVID-19 and consolidating social order.

#### 3.2.2. Diagnosis and Treatment Consolidation Pattern

**Figure 5:**
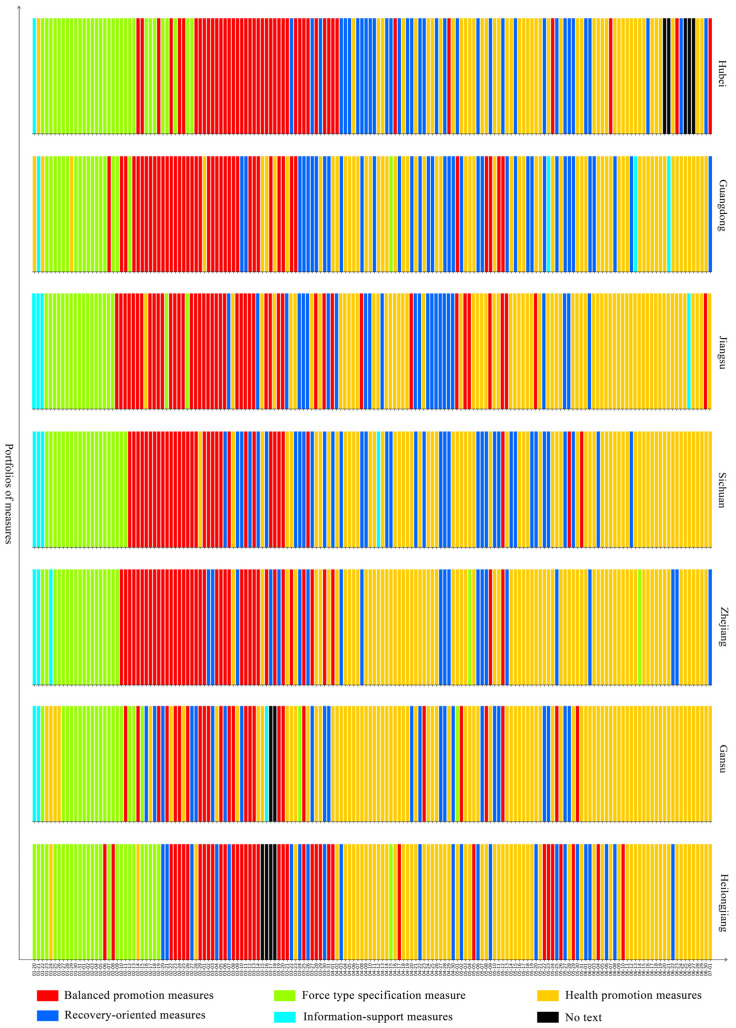

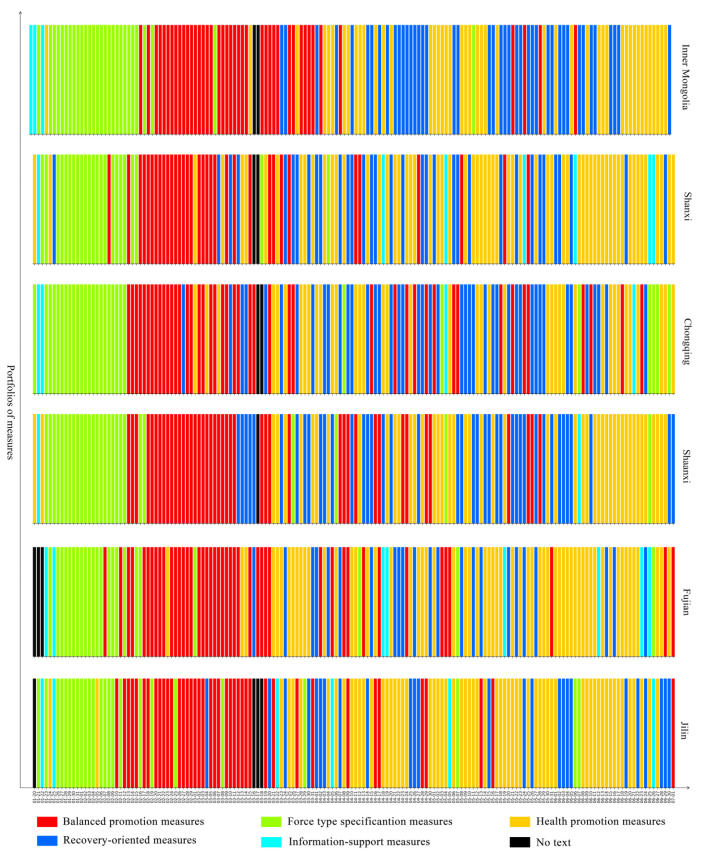
Diagnosis and treatment consolidation pattern.

A prominent feature of the diagnosis and treatment consolidation pattern is that it focuses on policies related to diagnosis and treatment and the flow of people in the later period of public health emergency management, including policies and measures such as improving diagnosis and treatment, improving prevention, diagnosis and isolation treatment, restricting the movement of people, controlling gathering activities, community prevention and control, monitoring and investigation, information support for prevention and control, and inter-regional cooperation. In the later stage of pandemic governance, it emphasised the development and consolidation of governance measures extended by the application of a policy portfolio. That is, when the national epidemic situation had been stabilised and the number of new cases detected every day had been brought to a low level—e.g., in the later stage of the control of the epidemic—the need to further consolidate the control results, reduce the risk of repeated outbreaks, and lay the foundation for promoting social recovery and economic development was emphasised. Areas following this pattern include Sichuan, Zhejiang, Gansu, Heilongjiang, Jiangsu, Guangdong, Shanxi, Shaanxi, Hainan, Inner Mongolia, Hubei, Jilin, and Fujian, for a total of 13 provinces. During the study period, balanced promotion measures were the main measures taken from 12 February to 20 March, for an average of 39 days; force-type specification measures were the main measures taken for an average of about 23 days, mainly focusing on the period from 20 January to 15 February; health promotion measures were the key measures taken for an average of 65 days, and were mainly concentrated in the period from 20 March to 1 July; recovery-oriented measures were the main measures taken for about 30 days; information-support measures were the main measures taken for about 4 days, presenting a scattered distribution (Please see Figure 5).

#### 3.2.3. Balanced Promotion Pattern

**Figure 6:**
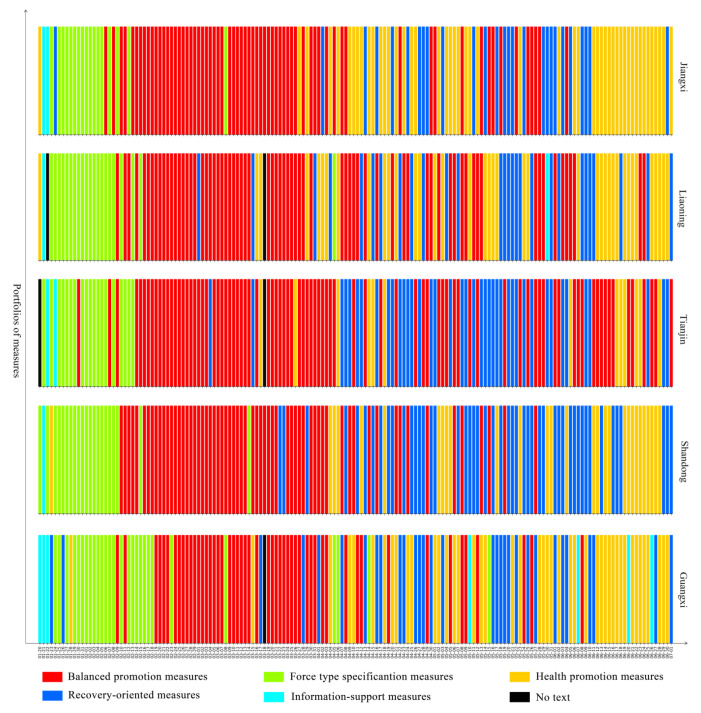
Balanced promotion pattern.

The most prominent feature of the balanced promotion governance pattern is that pandemic governance mainly focuses on command and coordination, recovery-related measures, support for Hubei province, the resumption of school, and the resumption of production. Through a combination of multidimensional measures, it is emphasised that the government should comprehensively adopt multidimensional governance methods and aim to promote the resolution of primary and secondary crises. This pattern includes Jiangxi, Liaoning, Tianjin, Shandong, and Guangxi, a total of five provinces. During the study period, balanced promotion measures were the main measures adopted in this pattern and were used for an average of 69 days, distributed in a concentrated manner from February 10 to 5 April and then at intervals from 7 April to 8 June. Force-type specification measures were the main measures taken for about 21 days, mainly focussing on the period from 22 January to 13 February. Health promotion measures were the main measures taken for an average of about 35 days, and were mainly in an interval distribution. Recovery-oriented measures were the main measures taken for an average of about 35 days, and were distributed at intervals from 8 April to 10 June. Information-support measures were the main measures taken for an average of about 3 days and showed a scattered distribution (Please see Figure 6).

#### 3.2.4. Quick-Adjustment Response Pattern 

**Figure 7:**
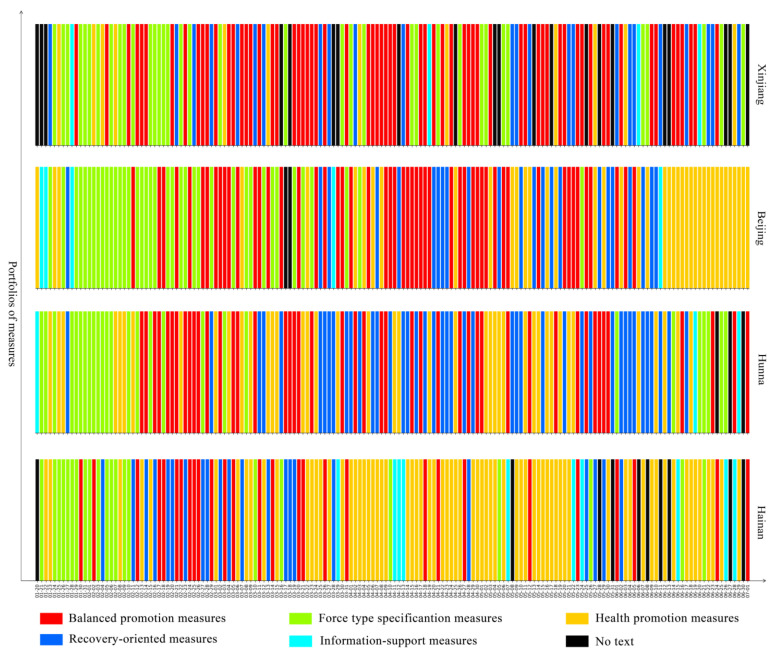
Quick-adjustment response pattern.

The most prominent feature of the quick-adjustment response pattern is that the speed of the policy updates and adjustments is very fast, making the policy response relatively flexible. Areas with this pattern emphasise the cross-promotion and comprehensive application of policies. The combination of multiple governance measures is more adaptable to complex governance scenarios and is conducive to improving the overall governance effect during the pandemic. This pattern includes the areas of Xinjiang, Beijing, Chongqing, and Hunan, for a total of four provinces. During the study period, balanced promotion measures were the main measures taken from around 12 February to 26 March, for an average of 53 days; force-type specification measures were the main measures taken for an average of about 33 days, mainly from 20 January to 12 February; health promotion measures were the main measures taken for an average of about 34 days and were mainly distributed at intervals; recovery-oriented measures were the main measures taken for an average of about 34 days and information-support measures were the main measures taken for an average of about 4 days, with both presenting a scattered distribution (Please see Figure 7).

#### 3.2.5. Recovery-Oriented Pattern

**Figure 8:**
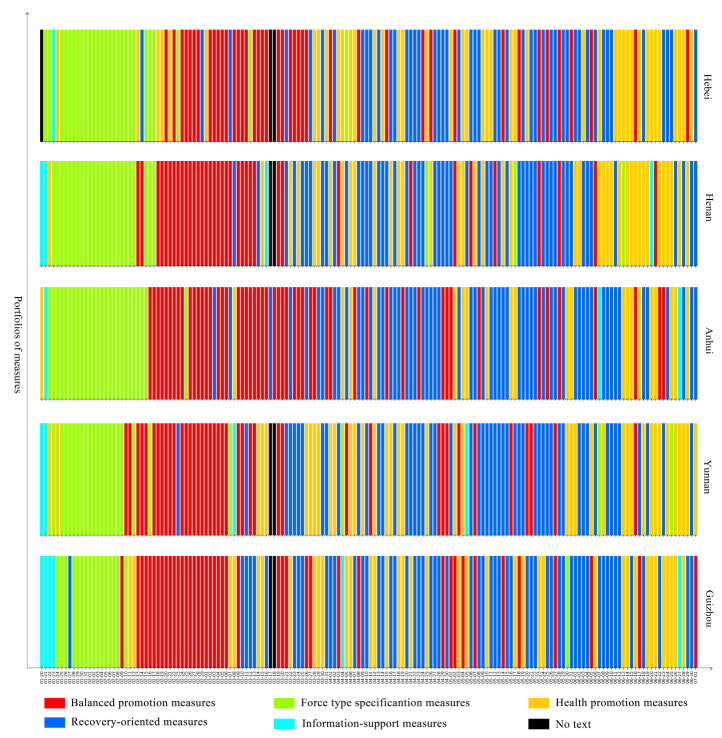
Recovery-oriented pattern.

The most prominent feature of the recovery-oriented pattern is that government governance in the later period emphasises the implementation of recovery measures, including measures such as resuming work, resuming production, and resuming school. These measures indicate a commitment to promoting social reconstruction and economic recovery through targeted measures. In the later period, the government’s focus is on the secondary crisis and it mainly aims to improve the public’s quality of life and satisfaction. Areas with this pattern include Guizhou, Yunnan, Anhui, Henan, and Hebei, for a total of five provinces. During the study period, balanced promotion measures were the main measures taken for an average of about 43 days from 15 February to 1 April; force-type specification measures were the main measures taken for an average of about 24 days, with these mainly being concentrated in the period from 22 January to 15 February; health promotion measures were the main measures taken for an average of about 37 days and were mainly distributed at intervals; recovery-oriented measures were the key measures used in this pattern for an average of 55 days, with these mainly being concentrated in the period from 21 March to 11 June; information-support measures were the main measures taken for an average of about 4 days, presenting a scattered distribution (Please see Figure 8).

## 4. Discussion

The goal of this paper is to represent the pandemic governance measures of different areas of the same country. To accomplish this, we applied text analysis and topic modelling methods to analyse the data collected from 28 provincial governments in mainland China from 20 January to 1 July 2020. In particular, this study reveals that the provincial governments’ responses to the pandemic were adjusted according to the realities of a process of dynamic evolution, showing obvious stage characteristics and differences between regions. The data collected can provide a basis for further summarising the five typical pandemic governance patterns of the Chinese regional governments’ responses to COVID-19. However, there may be many factors that can account for the different governance patterns followed across the same country.

### 4.1. China’s Experience and Its Contribution to Pandemic Governance

From the perspective of the dynamic changes in the use of China’s pandemic governance measures, China’s pandemic governance process has shown obvious stage differences and characteristics. The process of pandemic governance in China can be divided into three main phases.

In the first phase, the number of newly diagnosed cases nationwide increased rapidly, and the prevention and control situation was extremely severe. The number of new cases per day showed a continuous upward trend. On 12 February 2020, the highest number of confirmed cases of new coronary pneumonia in China was 15,152. At the same time, local governments initiated emergency responses to this major public health emergency and adopted various pandemic governance measures to jointly exert their efforts. Specifically, during this period popular science publicity measures were used most frequently in various provinces and cities and their level of use continued to be stable and high. This is because the novel coronavirus pneumonia pandemic was an unfamiliar event for the public. The government can improve the public’s pandemic awareness, as well as its awareness of prevention, through continuous scientific publicity so as to better achieve the goals of pandemic prevention and control. On the other hand, the most comprehensive, rigorous, and thorough national pandemic prevention and control strategies implemented at this stage were officially launched. Medical diagnosis and isolation treatment measures were used for the treatment and management of confirmed cases, effectively controlling the spread of infection. Surveillance and inspection measures focussed on an in-depth investigation of close contacts, targeted nucleic acid testing, and the implementation of the most stringent traffic quarantine to curb the development of the pandemic through preventing transmission.

In the second phase, the pandemic situation in Hubei Province was effectively controlled and the national pandemic situation generally became stable. From mid-March, new daily cases were controlled to the single digits; thus, the pandemic prevention and control efforts had clearly achieved important phased results. At this point, the government began to promote the resumption of work and production in an orderly manner, social and economic recovery, and the restoration of social order. At this time, the global pandemic spread rapidly, the number of confirmed cases increased hugely, and the world’s state of pandemic prevention and control was in crisis. Specifically, during this period compared with the previous stage, measures that focussed on resuming work and production began to be widely used and remained in place for a long stretch of time in most provinces and cities across the country, and their frequency of use continued to increase. The stable state of the pandemic at this point laid the foundation for promoting the restoration of a normal work order and production in society; at this stage, promoting the resumption of work and production was conducive to alleviating the socioeconomic problems that had been brought about by the pandemic. This allowed local governments to encourage economic recovery while increasing and stabilising the employment situation.

In the third phase, domestic outbreaks of COVID-19 were generally sporadic and the overall focus was on the prevention of cases imported from abroad. The pandemic prevention and control entered a positive trend and governments continued to consolidate the results of their prevention and control strategies. The national pandemic prevention and control entered a normalisation stage. In this stage, compared with the previous stage, the frequency of the implementation of the resumption of work and production measures, was further increased. The government further expanded the scope of the resumption of work and production measures across the whole society through financial support, tax reductions, and other means; improved the quality of the resumption of work and production measures; and promoted the rapid recovery and development of the economy and society. At the same time, reinstatement measures were widely used during this period and remained in place for a long time. The overall use of governance measures showed a phased change trend. The resumption of school was promoted in an orderly manner, and pandemic prevention and governance measures on campuses were strengthened to ensure the maintenance of a normal school order for students. At the information level, compared with the previous stage, the government strengthened the implementation of public opinion propaganda and social public opinion management measures, focussing on forming a positive public opinion in order to promote the restoration of social order and improve the government’s influence and credibility.

### 4.2. Analysis on Factors Affecting the Pandemic Governance Measures

From the perspective of the five typical pandemic governance patterns followed in China, this research revealed the provincial-level variation in the start, speed, and scope of pandemic governance measures used to fight COVID-19. As local governments must tailor their policy responses to local conditions, local governing contexts might be associated with the different choices in governance patterns adopted by provincial governments when facing similar crises. Figure 4, Figure 5, Figure 6, Figure 7 and Figure 8 show five types of policy distribution models: control pathogen pattern (the prominent feature of this policy distribution model is highlighting diagnosis and treatment across the whole period and the related measures of personnel mobility—that is, the mode of promotion of diagnosis and treatment used to deal with the primary crisis); diagnosis and treatment consolidation pattern (this pattern emphasises the implementation of measures related to diagnosis, treatment, and personnel mobility restriction); balanced promotion pattern (this pattern focuses on command and coordination and recovery-related measures, including command and coordination, support for Hubei province, the resumption of school, the resumption of production, and other pandemic governance measures); quick-adjustment response pattern (where the prominent feature is that the speed of policy updates and adjustments is fast, making the policy response relatively flexible); recovery-oriented pattern (strengthens the extent and scope of recovery measures taken, including measures such as resuming work, resuming production, and resuming school).

#### 4.2.1. Factors Leading to Following a Controlling Pathogen Pattern

Shanghai is the only area that adopted the pathogen-control pattern to fight COVID-19. Previous studies have demonstrated that GDP per capita has a positive association with governmental economic stimulus [7]. However, as a city with a high level of GDP per capita, this city was able to shoulder the burden caused by the pandemic and by the stringent measures taken. The pandemic situation in Shanghai is not positive, with many imported cases; as of 24:00 on 1 July, 371 confirmed imported cases had been reported in total. Furthermore, Shanghai’s scale of foreign trade is greater compared to that of other regions, which means that it has a higher risk level in terms of economic restoration. Additionally, the high elderly population dependency ratio and large population density in Shanghai have contributed to the pandemic instability in this region, putting even more pressure on the Shanghai government to contain the pandemic. These factors together have increased the projected burden on the government and prompted the Shanghai government to adopt more stringent measures. 

#### 4.2.2. Factors Leading to Following a Diagnosis and Treatment Consolidation Pattern

The diagnosis and treatment consolidation pattern was mainly adopted in provinces that experienced higher numbers of COVID-19 outbreaks and saw more negative impacts on the local economy—i.e., the Hubei province. The average number of confirmed cases in these provinces is much higher than that in other regions, reaching 8200 cases. Bastián et al. claimed that the higher number of confirmed cases might be associated with a slower implementation of suppression interventions [12]. However, in this study our results concerning the governing context of the diagnosis and treatment consolidation pattern could not verify this finding. Moreover, the high population mobility in these areas has increased the urgency of the need to contain the pandemic. These particular local conditions have strengthened the necessity of implementing more stringent measures. However, the decline in the GDP index (9.3%) in these provinces is the largest of all regions, which has also increased the urgency of the need to implement economic restoration measures. These heavy social and economic burdens have prompted these governments to slightly decrease their number of stringent measures to balance the containment of the pandemic and people’s livelihoods.

#### 4.2.3. Factors Leading to Following a Balanced Promotion Pattern

The balanced promotion pattern was mainly adopted in governing contexts where the pressure to control the pandemic and the urgency of economic restoration were at fairly equal levels. On the one hand, in these provinces the insufficient number of hospital beds increased pressure on the public health system, meaning that prevention, diagnosis, and treatment measures were necessary. However, the low population mobility in these areas might have acted as a buffer against the pressure of containing the pandemic. On the other hand, these provinces’ high economic development might have acted as a buffer against the economic burden caused by the pandemic and, in turn, contributed to there being only a medium level of urgency regarding reconstructing the local economy. Due to the relatively balanced levels of urgency of the containment of the pandemic and the restoration of the economy, these governments’ responses to COVID-19 were relatively equal across each epidemic governance dimension.

#### 4.2.4. Factors Leading to Following a Quick-Adjustment Response Pattern 

The quick-adjustment response pattern was mainly adopted in provinces that had an insufficient number of hospital beds, a small elderly population dependency ratio, a low population density, and a low level of mobility. The insufficient number of hospital beds in these areas may have caused social anxiety among people, and young people on a large scale in these areas may have been exposed to rumours through social media, which might have exacerbated social panic. These factors prompted the governments in these areas to emphasise risk communication.

#### 4.2.5. Factors Leading to Following a Recovery-Oriented Pattern

Previous studies have demonstrated that the total number of COVID-19 cases is positively related to the extent of economic stimulus [7]. In the provinces that adopted the recovery-oriented pattern, the average number of total COVID-19 cases was 371, fewer than that in any other region. Sufficient hospital beds, low human mobility, and low population density all proved conducive to the stability of the pandemic situation, making it possible for the government in these areas to prioritise economic restoration. Additionally, the small scale of foreign trade in these areas decreased the risk of governments implementing economic restoration measures, thus encouraging them to take an additional step towards the reconstruction of the economy.

## 5. Conclusions

This study advances research on the pandemic governance measures implemented in Chinese provincial governments and offers a summary of regional governments’ overall pandemic governance patterns. It also tracks the evolution of regional government responses, analysing the differences between the regional government responses by measuring the extent and intensity of 23 measures adopted by every provincial government. Our results indicate that different provinces targeted their selection of the time points when pandemic governance measures needed to be used, the time period for their continuous use, and the combination of measures used according to the pandemic development and economic conditions in their local area. The provincial pandemic governance patterns adopted in China can be divided into a pathogen-control pattern, a diagnosis and treatment consolidation pattern, a balanced promotion pattern, a quick-adjustment response pattern, and a recovery-oriented pattern. The results of the discussion show that pandemic severity, economic development, public health services, and population structure may have an impact on pandemic governance measures. However, the discussion of the factors influencing the choice of pandemic governance pattern used, reveals that a more thorough analysis can be made through the judgment and description of the pattern’s diagrams, which may be detrimental to the accuracy of the analysis. These limitations will continue to be addressed in future research.

## Figures and Tables

**Table 1:** Coding book.

Selective Coding Index	Axial Coding Index (Measures)	Open Coding Index (Keywords)
Information	Information update	CCTV; Prevention; Diagnosis; Human transmission; Personal protection
	Popular science dissemination	Publicity; Popular science; Authoritative experts; Guidance; Give a cue
	Public opinion propaganda	CCTV; Rumour; Come on; Win; Spread rumours
Supervision	Market supervision	Price increase; Price; Product supply; Market supply; Price of medical commodity
	Supervision of popular behaviour	Cooperate; Prevention of obstruction; Conceal facts; Deliberate concealment; Conceal illness
	Supervision of official performance	Perform duties; Inadequate prevention and control; Responsibility implementation is not in place; Dereliction of duty; Underreport
	Material supervision	Material supervision; Mask; Medical use; Materials; the Red Cross Society
Diagnosis and treatment	Diagnosis; isolation; treatment	Isolation; Clinical symptoms; Medical observation; Designated hospitals; Treat and cure
	Improve diagnosis and treatment	Nucleic acid detection; Chinese medicine; Hospital of traditional Chinese medicine; Virus strain; test kit
	Improve prevention	Vaccine research; Vaccination; Scientific research; Vaccine; Laboratory
	Support of Information-based diagnosis and treatment	AI diagnosis; Teleconsultation; Green channel for medical consultation; Online medical treatment
Recovery	Resumption of school	Students; Term begins; Colleges and universities; Teaching; Online teaching
	Resumption of work and production	Resumption of work; Production; Resumption of production; Economy; (of laid-off employees) return to the original job
Security	Financial guarantee	Funds; Medical insurance; Guarantee; Financial bottom; Capital
	Self-material support	Mask; Medical use; Materials; Protective clothing; Donation
Command and coordination	Command and coordination	Research deployment; Instructions; Deploy; Leading group; Inspection and guidance
	Support for Hubei Province	Mobilisation; Medical team; Expedition; Protective clothing; Donation
Movement of people	Restrict the movement of people	Go out; Home; Outage; Return home
	Control of gathering activities/places	Public places; Cancellation of aggregated activities; Suspension of opening to the outside world; Start closing; Scenic spot
	Community prevention and control	Community; Community residents; Housing estate; Streets; Residents’ committee
	Monitoring and investigation	Close contact; Nucleic acid detection; Check; Epidemiological investigation; Traffic quarantine; Virus detection
	Support of information-based prevention and control	Green code; Gan code; Health code; Five-colour map of pandemic situation; Pandemic information collection

## Data Availability

The data presented in this study are available on request from the corresponding author. The data are not publicly available due to the fact that the data are also being used in an ongoing study.
